# Biodegradable biocomposite pots reinforced with mercerized sugarcane bagasse for sustainable agriculture and plastic waste mitigation

**DOI:** 10.1038/s41598-025-01419-y

**Published:** 2025-05-17

**Authors:** Manar E. Elashry, Elsayed G. Khater, Samir A. Ali

**Affiliations:** Agricultural and Biosystems Engineering Department, Faculty of Agriculture, Benha University, Banha, Egypt

**Keywords:** Biodegradable pots, Mercerization, Natural fillers, Physical properties, Mechanical properties, Plant growth, Sustainable agriculture, Ecology, Environmental sciences

## Abstract

Biodegradable cultivating pots were fabricated using biocomposites reinforced with fillers such as sugarcane bagasse, compost, peat moss, vermiculite, and activated carbon. This study investigates the impact of mercerization pretreatment on the physical and mechanical properties of pots made with palm wax and Lanette wax matrices. Pretreatment improved fiber-matrix adhesion, reducing water absorption (17–11%) and void content (3.4–1.9%), while increasing tensile strength by 4%. Greenhouse experiments demonstrated enhanced growth performance in Anaheim chili pepper plants grown in treated pots, with specific vegetative indices reaching up to 99.75% and yield improvements of up to 26.6 peppers per plant. The findings emphasize the potential of integrating natural fillers and pretreatment techniques to develop sustainable alternatives to traditional plastic pots, contributing to eco-friendly agricultural practices.

## Introduction

The escalating production of bio-based polymers reflects a global shift toward sustainable material solutions, with output soaring from 3.5 million tons in 2011 to a projected 12 million tons annually by 2020^1^. However, this progress pales compared to the staggering 235 million tons of petrochemical-based polymers produced yearly, underscoring the urgent need for innovative alternatives to mitigate plastic waste and greenhouse gas emissions. In response, green composites have emerged as a beacon of ecological innovation, particularly in applications such as biodegradable cultivating pots that merge sustainability with functionality.

Biodegradable pots eliminate the need for container disposal post-transplantation, reducing landfill waste while enriching soil ecosystems^2^. By integrating natural fillers like sugarcane bagasse, compost, and activated carbon, these pots achieve enhanced water management, structural stability, and nutrient delivery—attributes vital for supporting plant growth^3^. Additionally, mechanical properties such as tensile strength and elongation, critical for withstanding environmental stresses, are further refined through pretreatment processes like mercerization, which strengthens the filler-matrix bond by removing lignin and hemicellulose^4^.

Sugarcane bagasse, a byproduct of the sugar industry, epitomizes the potential of agro-industrial residues as sustainable reinforcements in composites. With high tensile strength, low density, and biodegradability, bagasse fibers serve as eco-friendly substitutes for synthetic fillers^5^. Compost, revered for its organic richness, enhances soil fertility and water retention^6^, while vermiculite and peat moss improve aeration and root penetration^3^. Activated carbon, with its superior adsorption capacity, contributes to the composite’s ability to retain nutrients and moisture^7^. Together, these fillers create a multifunctional blend that supports plant growth while reducing the environmental footprint.

Adopting biodegradable pots addresses two critical challenges: reducing reliance on plastics and enhancing agricultural efficiency. These pots can be directly planted into the soil with crops, where they naturally decompose, enriching the soil with organic matter^2^. Physical attributes such as water absorption, density, and void space play integral roles in water retention and root aeration, while mechanical properties such as tensile strength ensure stability and longevity^4^. By optimizing these characteristics through innovative filler combinations and treatments, biodegradable pots hold the potential to revolutionize sustainable agriculture.

This research evaluates the physical, mechanical, and ecological performance of cultivating pots made from palm and Lanette wax matrices, incorporating fillers like sugarcane bagasse, compost, and activated carbon, with and without mercerization pretreatment. By examining parameters such as water absorption, tensile strength, and plant growth, this study contributes to the progress of eco-friendly alternatives to traditional plastic-based pots, offering solutions that align with global sustainability goals and advance agricultural innovation.

## Materials and methods

**Fig. 1:**
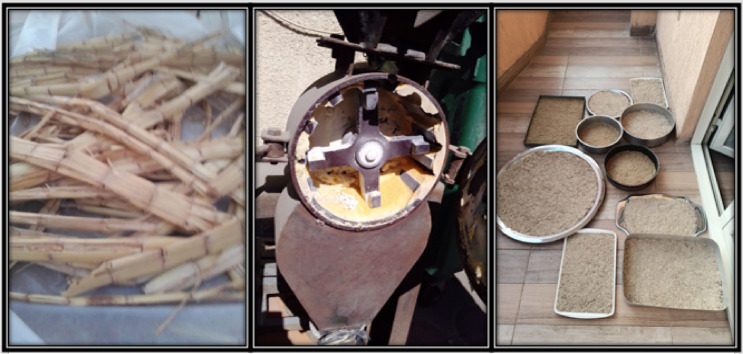
Preparation of sugarcane bagasse for biodegradable cultivating pots.

The biodegradable cultivating pots were constructed as biocomposites, utilizing various filler reinforcement materials, including sugarcane bagasse obtained from local farms, subjected to grinding, washing, and drying processes to produce the powders depicted in Fig. 1, Various fillers, such as compost, peat moss, vermiculite, and activated carbon, were procured from local suppliers. Sorbitol was incorporated as a plasticizer to facilitate the homogenous blending of ingredients, while sodium hydroxide was obtained from a local provider for the mercerization pretreatment of sugarcane bagasse. These materials were combined with two binding agents, namely Palm (P) and Lanette wax (L), as well as the impact of sugarcane bagasse mercerization pretreatment with 1 M NaOH to create biodegradable biocomposite matrices for use in cultivation pots, as outlined in Table 1.

**Table 1:** Experimental layout description of candidate cultivating pot materials used in the investigation.

Material	Abbreviation	Description
Palm wax-based pot	P	Fabricated pots made from palm wax (Stearic acid) and filler materials without pretreatment of sugarcane bagasse
Palm wax-based pot with pretreatment	PW	Fabricated pots made from palm wax and filler materials with 1 M NaOH pretreatment of sugarcane bagasse
Lanette wax-based pot	L	Fabricated pots made from Lanette wax (cetyl alcohol) and filler materials without pretreatment of sugarcane bagasse
Lanette wax-based pot with pretreatment	LW	Fabricated pots made from Lanette wax (cetyl alcohol) and filler materials with 1 M NaOH pretreatment of sugarcane bagasse

### Apparatus employed in the fabrication of cultivating pots

**Fig. 2:**
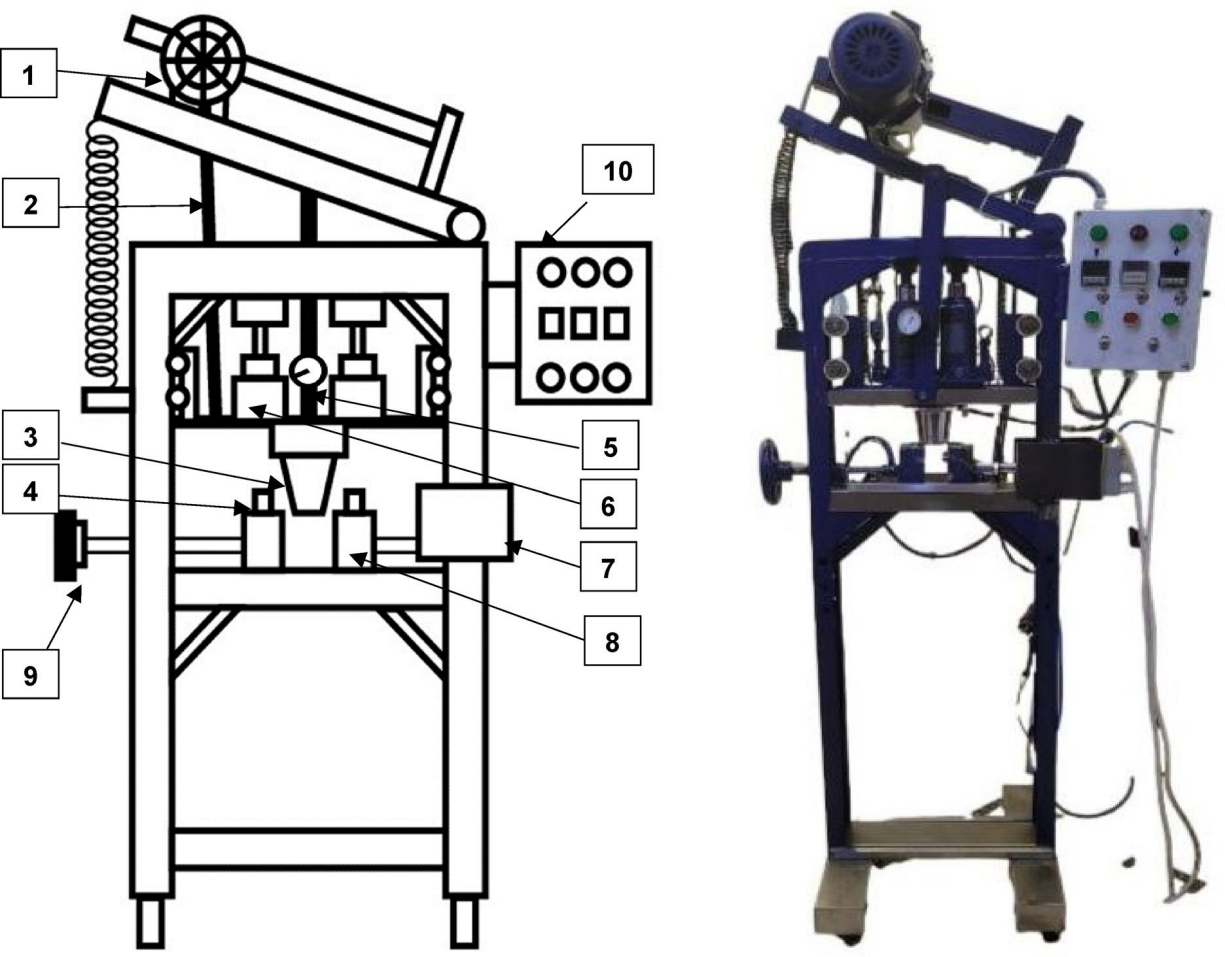
Configuration of the assembled machine used in the fabrication of cultivating pots. 1—1.5 hp 3-phase geared motor, 2—Crank mechanism, 3—forming mold, 4—Heaters, 5—Pressure gauge, 6—Hydraulic bottle jack, 7—Pneumatic clamping cylinder, 8—Pouring mold component, 9—Screw wheel clamping, 10—Control panel.

The apparatus utilized in the fabrication of cultivating pots is depicted in Fig. 2. As illustrated in the layout, it comprises a 1.5 hp 3-phase geared motor mounted on a crank mechanism to actuate the ram of a hydraulic bottle jack, equipped with a pressure gauge, on a stationary forming mold. The pouring mold, divided into two parts, is equipped with heaters, and mounted on a movable clamping pneumatic and screw wheel. The entire mechanism is adjusted by a control panel.

**Fig. 3:**
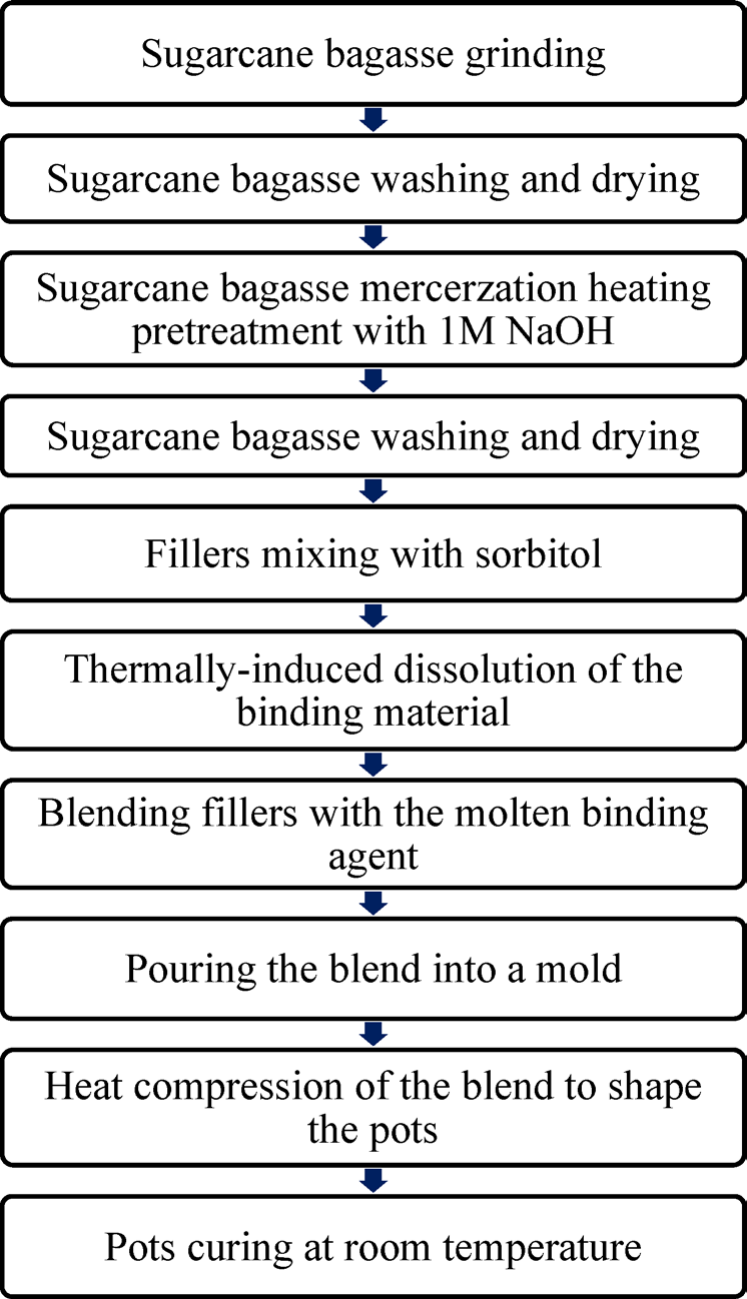
Flow chart outlining the fabrication methodology for cultivating pots.

Composite pots were fabricated using a melt-blending and compression molding process, as illustrated in the flowchart in Fig. 3. In this process, palm and Lanette waxes were heated and homogenized with predetermined filler proportions, consisting of 35 wt% sugarcane bagasse, 35 wt% compost, 10 wt% peat moss, 10 wt% vermiculite, and 10 wt% activated carbon. The wax-to-filler ratio was maintained at 5:4 (wt%). The blended mixture was then poured into a pre-prepared steel mold and subjected to hot pressing under a pressure of 160 bars at 60 °C to achieve the desired pot structure.

In the case of mercerization pretreatment specimens, sugarcane bagasse underwent drying, grinding, and thorough washing. Delignification was achieved through bleaching under pressure at 1.5 Kpa and 60 °C with 1 M NaOH for 30 min. The resulting black liquor and thick slurry were rinsed multiple times to achieve a neutral pH and then microwave-dried. The wet weight of the fibers underwent intermittent microwave heating at 600 W for 3 min initially to prevent overheating, followed by subsequent heating to ensure uniform drying for 1 min until a constant weight was reached, as outlined by^8^.

### Physical properties of cultivating pots

#### Water absorption of the cultivating pots (WA)

The water sorption (WA) by the composite specimens was measured according to ASTM D570^9^ by taking the initial weight (W_0_) before immersing them in a beaker filled with distilled water at 23 °C for 24 h, then removing out, surface drying using tissue paper to remove the excess water up to reach equilibrium state and recording their weight (W) frequently using a digital balance with a 0.1 mg accuracy. Average value was calculated considering three replicates of square specimens (20 × 20 mm). The water absorption was calculated according to the following formula according to^10^.

1 $$\:\text{W}\text{A}\:\left(\text{\%}\right)\:=\frac{\text{W}-\text{W}0}{\text{W}0}\times\:\:100\:\text{\%}$$

Where: WA is the water sorption, %, W_o_ is the initial weight (g), and W is the weight after immersion for 24 h. (g).

#### Density of the cultivating pots

The density *ρ* of composites in respect of weight fraction is attained using the following generalized equation for an arbitrary number of constituents^11^.

2 $$\:{\uprho\:}=\frac{1}{\sum\:_{i=1}^{n}(Wi/\rho\:i)}$$

Where: W_i_ represents the weight fraction, and ρ_i_ denotes the density of the filler constituent (g. cm^− 3^), as specified in the manufacturer’s data sheet (Table 2).

**Table 2:** Density of each constituent for cultivating pots.

Filler type	Density (g/cm^3^)
Sugarcane bagasse	1.25^12^
Compost	0.655
Peat moss	0.5
Activated carbon	2.1
Vermiculite	0.16
Palm wax	0.85
Lanette wax	0.82

#### Porosity or voids (V)

As the theoretical density (ρ_ct_) may not agree with the actual density (ρ_ce_) due to voids existence in the composites, the actual density was determined experimentally through a simple gravimetrical water immersion technique and the voids were determined by the ratio of difference between densities to the theoretical one as given as follows:

3 $$\:\text{V}\:=\frac{\rho\:ct-{\uprho\:}\text{c}\text{e}\:}{{\uprho\:}\text{c}\text{t}\:}$$

According to (Werber 1980), the higher void content, the greater susceptibility to water dispersion, consequently affecting the strength properties. The void content may range between less than 1% for the good composite and 5% for the poor one^11^.

### Mechanical properties of cultivating pots

#### Tensile strength of the cultivating pots

Seedling pots face numerous forces emanating from the inside of the pots due to plant growth and handling in a greenhouse setting^13^. The mechanical properties of the fabricated biocomposite cultivating pots were evaluated following ISO 527- 4^14^, a widely adopted standard for fiber-reinforced composites. The applied compression force during the fabrication process influences the tensile strength of the pots, as previously confirmed^13^. This effect is attributed to particle rearrangement, which leads to a more tightly packed structure, reducing porosity and enhancing the tensile strength of the formed sheet.

Most prior studies on biodegradable pots have involved the preparation of sheets in advance for mechanical testing^10,15^. However, in this study, samples were directly extracted from the walls of the fabricated pots to exploit the effects of high fabrication compression on mechanical strength. The dimensions of the pot walls were well-suited for sample preparation in accordance with ISO 527-4 specifications.

Tensile strength serves as a key indicator of internal bonding and overall pot stability. The fabricated pots were sectioned into small specimens with a fixed thickness of 5 mm, corresponding to the initial pot wall thickness and within the limits specified by ISO 527-4. Uniaxial tensile testing was performed on rectangular specimens (50 mm of gauge length× 25 mm of width) cut from the pot walls using a universal testing machine (UTM) equipped with a 5 kN load cell. The tests were conducted at a crosshead extension speed of 2 mm/min under ambient conditions, in compliance with ISO 527- 4 requirements.

4 $$\:\text{T}\text{e}\text{n}\text{s}\text{i}\text{l}\text{e}\:\text{s}\text{t}\text{r}\text{e}\text{n}\text{g}\text{t}\text{h}\:\left(\text{T}\right)\:=\frac{\text{F}}{\text{A}}$$

Where F and A are denoted as maximum applied force (N), cross-sectional area of the sample (mm²). Triplicates of each composite were tested, and the average value is reported.

#### Elongation at break for the cultivating pots (ε)

Elongation of the composites was detected at the break point at the ultimate tensile strength.

5 $$\:\text{E}\text{l}\text{o}\text{n}\text{g}\text{a}\text{t}\text{i}\text{o}\text{n}\:\left({\upepsilon\:}\right)\:=\frac{\Delta L}{{L}_{o}}\times\:\:100\text{\%}$$

Where: △L is elongation at tensile break (mm), and L_o_ is the initial gauge length (mm).

#### Tensile modulus of elasticity at break for the cultivating pots (E)

The tensile modulus of elasticity at the break for the cultivating pots was calculated by dividing the tensile strength by the elongation, as per the following equation:

6 $$\:\text{E}\text{l}\text{a}\text{s}\text{t}\text{i}\text{c}\:\text{m}\text{o}\text{d}\text{u}\text{l}\text{u}\text{s}\:\left(\text{E}\right)\:=\frac{\text{T}}{{\upepsilon\:}}=\frac{(F/A)}{(\Delta L/{L}_{o})}$$

### Assessment of biodegradable pots on pepper plant growth

Anaheim chili peppers (*Capsicum annuum*) were obtained from the farm of the Faculty of Agriculture, Benha University, and cultivated in the pots under controlled greenhouse conditions. In a completely random experiment, each of the four types of composites was represented by five pots. These were compared against a commercial plastic pot, which was removed and directly transplanted into the soil (C-pot), acting as the control.

**Fig. 4:**
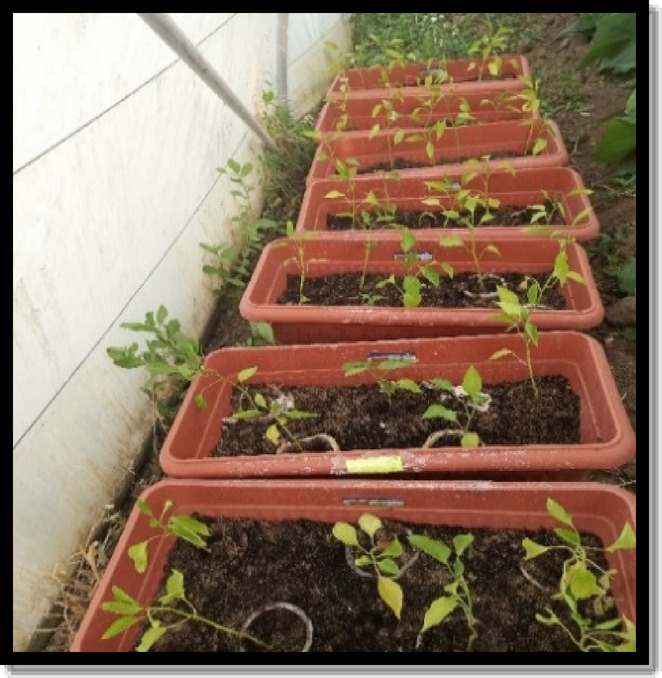
Pepper planting into the pots in a greenhouse experiment.

The productivity of these pots on the pepper plants was assessed, including vegetative growth and yield parameters, after approximately three months of growing, using one-way ANOVA to determine statistical significance (Fig. 4).

Vegetative growth parameters were assessed, such as shoot height, root length, number of branches, leaves per plant, and Specific Vegetative Index (SVI). Additionally, yield parameters, encompassing fresh plant weight, plant length, plant diameter, and the number of peppers per plant, were evaluated to gauge the overall performance of the pots in supporting pepper plant growth^16^.

7 $${\text{SVI }}={\text{ }}\left[ {{\text{ }}\left( {{\text{Lr}}} \right)+{\text{ }}\left( {{\text{Ls}}} \right)} \right]{\text{ }} \times {\text{ }}\left( {{\text{GP}}} \right){\text{ }}\%$$

Where Lr is the mean root length, Ls is the mean shoot length, and GP is the percentage of seed germination (%)^17^. The germination percentage (GP) was recorded as 100%, as all planted pots exhibited successful germination and seedling growth.

## Results and discussion

### Physical properties of cultivating pots

#### Shape characteristic of the cultivating pot

**Fig. 5:**
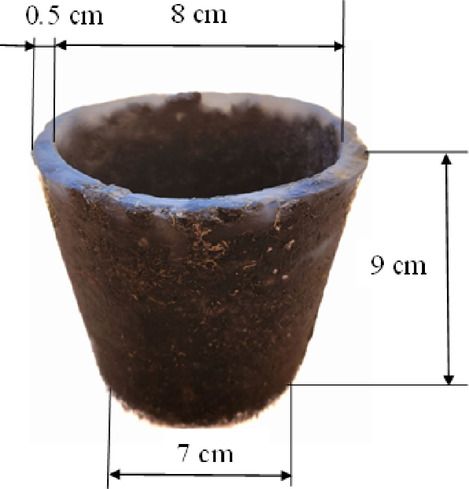
Dimensions of fabricated biodegradable planting pot.

The cultivating pots were distinguished by their conical frustum shape as illustrated in Fig. 5, featuring dimensions of upper and lower diameters, height, and thickness measuring 8, 7, 9, and 0.5 cm, respectively. The weight of these pots varied within the range of 170 to 200 g.

#### Water absorption (WA)

As presented in Table 2, the water absorption values for LW, L, PW, and P-pot were measured at 15%, 17%, 11%, and 14%, respectively. The highest absorption (17%) was recorded for the L-pot, whereas the PW-pot exhibited the lowest absorption at 11%. This variation in water absorption can be attributed to differences in filler composition and interfacial adhesion within the composite matrix. The reduction in water absorption following pretreatment is associated with enhanced fiber–matrix bonding, which decreases porosity and limits water penetration, consistent with previous findings^18^.

The water uptake behavior of biocomposite pots, particularly those formulated with natural fillers, remains inconsistent despite pretreatment. In the case of epoxidized soybean oil (ESO)-modified composites, the absence of crosslinking and grafting mechanisms involving end-terminated poly(ethylene glycol) diglyceryl ether (PEG) derivatives results in increased hydrophilicity and compromised structural integrity upon moisture exposure^19^. The incorporation of waxes as crosslinking agents provides an alternative approach, acting as a hydrophobic barrier that mitigates rapid water uptake while facilitating the controlled release of nutrients over time.

Moisture absorption in composite materials is a function of multiple parameters, including fiber orientation, void content, fiber volume fraction, interfacial adhesion, and environmental conditions. The presence of hydrophilic constituents such as cellulose, hemicellulose, and lignin in agricultural residue-based pots contributes to their affinity for water. However, when crosslinked with hydrophobic polymers, these materials exhibit absorption behavior consistent with the Fickian diffusion model^20^. The observed trends in water absorption highlight the critical role of material formulation and processing techniques in determining the long-term performance and stability of biodegradable pots.

#### Density

As presented in Table 2, the P-pot exhibited a higher density (0.71 g/cm³) compared to the L-pot (0.69 g/cm³). This difference corresponds to the inherent densities of the waxes used in the formulations, where palm wax (0.85 g/mL) has a greater density than hexadecanol (0.82 g/mL). The observed density variation suggests that the selection of wax type influences the overall compactness and structural integrity of the composite material.

Apparent density is a critical parameter influencing the decomposition behavior of biodegradable pots in the soil. Lower-density materials facilitate the penetration of moisture and nutrients through the pot walls, thereby enhancing microbial activity and accelerating decomposition^10^. Consequently, the density of the fabricated pots plays a significant role in determining their degradation rate and overall performance in soil environments.

#### Porosity (voids)

As delineated in Table 3, the void percentages for LW, L, PW, and P-pot were documented as 2.6%, 3.4%, 1.9%, and 2.9%, respectively. The reduction in voids following pretreatment may be attributed to the removal of lignin and hemicellulose, as well as the enhancement of adhesion between the fiber and the adhesive matrix.

**Table 3:** Physical properties of various fabricated cultivating pots.

Label	Water absorption (%)	Voids (%)	Density (g/cm^3^)
P	14 ± 1^B^	2.9 ± 0.1^B^	0.712575888 ± 0^A^
PW	11 ± 1^C^	1.9 ± 0.1^D^	0.712575888 ± 0^A^
L	17 ± 1^A^	3.4 ± 0.1^A^	0.693427782 ± 0^A^
LW	15 ± 1^AB^	2.6 ± 0.1^C^	0.693427782 ± 0^A^

Means that do not share a letter differ significantly at *p* < 0.05 (Tukey’s HSD).

### Mechanical properties of cultivating pots

It is crucial to evaluate the strength at break of these containers, considering their vulnerability to greenhouse conditions, including handling, crushing, and shipping. During plant growth, there is an emanating force from inside the container, attempting to penetrate the walls outwards due to the weight of saturated soils or roots. This assessment is vital to ensure their resilience under such circumstances^21^.

**Fig. 6:**
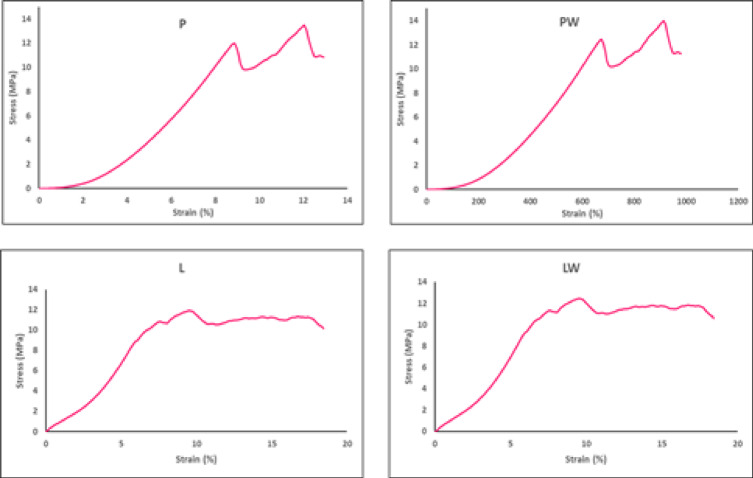
Typical stress-strain curve for different pot varieties.

Figure 6 illustrates the typical pattern of stress-strain curves obtained from tensile tests conducted on various biodegradable pots. Tensile stress values at break for P and L-pots are reported in Table 4 as 13.46 and 11.88 MPa, respectively. The application of mercerization pretreatment led to an increase in tensile stress to 14 and 12.45 MPa for P and L-pots, respectively. The improvement in tensile strength parallels the beneficial increment observed in textile and cardboard-based pots, as indicated by^13^. This enhancement is attributed to the disruption of crystalline cellulose and the partial elimination of hemicellulose, lignin, and other substances, increasing reaction sites and rearrangement with enhanced surface roughness of the fiber structure. Furthermore, this increase is consistent with the findings of^22^.

**Table 4:** Mechanical tensile properties for the pots.

Pots label	Elongation (%)	Tensile stress (MPa)	Tensile modulus (MPa)
P	12.04508493 ± 0.011^A^	13.46652222 ± 0.014^B^	111.8009736 ± 0.010^B^
PW	11.50508493 ± 0.013^B^	14.00652222 ± 0.011^A^	121.742015 ± 0.019^A^
L	9.591391059 ± 0.011^C^	11.88592692 ± 0.010^D^	123.9228684 ± 0.203^D^
LW	10.19059106 ± 0.010^C^	12.45592692 ± 0.012^C^	122.2296808 ± 0.104^C^

Means that do not share a letter differ significantly at *p* < 0.05 (Tukey’s HSD).

The tensile modulus values for P, PW, L, and LW were measured at 111.8, 121.74, 123.922, and 122.22 MPa, respectively. Concurrently, the corresponding elongation values were 12.04%, 11.5%, 9.59%, and 10.19%, respectively. PW-Pots demonstrated improved tensile modulus, and elongation values decreased slightly, reflecting the trade-off between stiffness and flexibility.

The incorporation of natural fillers such as sawdust and wood flour has been shown to enhance the tensile strength of biocomposite materials. These fillers, derived from renewable resources, contribute to improving thermo-mechanical properties and reducing water sensitivity while maintaining biodegradability. Their addition also enhances matrix homogeneity by minimizing phase separation, thereby increasing the interaction between composite components^19^.

The tensile strength (TS) of hydrolyzed soy protein isolate-based composites has been reported to increase from 18.15 MPa to 19.89 MPa with the addition of 6% urea. However, exceeding the critical interfacial concentration threshold leads to a decline in tensile strength, decreasing from 18.42 MPa to 15.85 MPa^23^. This highlights the importance of optimizing filler and additive concentrations to achieve desirable mechanical properties^20,24^.

Both the fibers and the binding matrix significantly influence tensile stress. A study investigating the use of discarded textiles (cotton and polycotton) and paper waste (newspaper and corrugated cardboard) as substrates, along with different binding agents (molasses, sodium alginate, and cornstarch), demonstrated that cornstarch was the most effective binder for improving tensile strength. At a concentration of 20% (dry weight basis per sheet), cornstarch achieved tensile strengths of 2.19 MPa, 2.93 MPa, 5.47 MPa, and 6.20 MPa for polycotton, cotton, corrugated cardboard, and newspaper sheets, respectively, moreover, cornstarch and sodium alginate proved effective in enhancing tensile properties, whereas molasses did not yield significant improvements^13^.

Pretreatment methods, particularly alkali treatment, have been shown to significantly enhance tensile strength. An optimal tensile strength was achieved by treating cotton, polycotton, and newspaper sheets with 5% NaOH, with newspaper sheets reaching a maximum tensile strength of 4.33 MPa, followed by cotton sheets at 2.73 MPa. Soaking these materials in 5% NaOH for 5 h improved tensile properties by removing impurities and reducing surface defects. However, for corrugated cardboard sheets, alkali treatment did not enhance tensile strength^13^, indicating that its effectiveness depends on the filler type and structural composition.

In the current study, the tensile strength of the fabricated pots ranged from 11.9 MPa to 14 MPa, exceeding the values reported in most previous studies, particularly those utilizing starch and sodium alginate-based composites, as shown in Table 5. However, the tensile strength remained lower than that of pots reinforced with urea-formaldehyde composites^23^. Despite incorporating straw fiber and hydrolyzed soy protein as biodegradable components, urea-formaldehyde is a fossil fuel-derived, non-biodegradable resin commonly used in the particleboard industry. The obtained tensile strength values were comparable to those of biodegradable pots fabricated using starch and polybutylene adipate-co-terephthalate (PBAT)^25^.

**Table 5:** Tensile strength of some biodegradable pots.

Biomass raw material	Binding matrix	Tensile strength (MPa)	Reference
-	Thermoplastic starch produced from native cassava starch & glycerol with a biodegradable polymer such as: (poly butylene adipate-co-terephthalate) (PBAT)	12.4–13.2	^25^
Protein hydrolysate (Pr.Hyd) from leather industry wastes, saw dust & wood flour	Poly (ethylene glycol) diglyceryl ether (PEG) or epoxidized soybean oil (ESO), ethylene diamine (EDA)	4–13	^19^
Straw fiber	Hydrolyzed soy protein isolate/urea/formaldehyde	15.85–19.89	^23^
(Paper substrates: newspaper & corrugated cardboard) combined with textile waste (cotton & polycotton)	Molasses Sodium alginate Cornstarch	2.73–6.20	^13^
Paddy straw with alkali and autoclave – treatment & without treatment	Corn starch, boric acid, and glycerol.	1.96–6.82	^26^

### Assessment of biodegradable pots on pepper plant growth

**Fig. 7:**
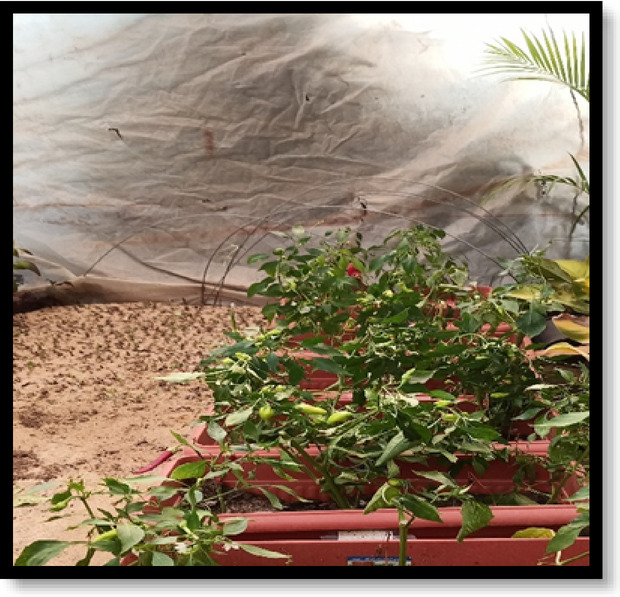
Biodegradable pots with pepper plants in a greenhouse experiment.

Five biodegradable pots of each of the four types, along with a control, were employed for cultivating pepper plants, as illustrated in Fig. 7, to evaluate their compatibility with environmental ecology. The assessed characteristics were categorized into two parameters: vegetative growth parameters and yield parameters.

### Vegetative growth parameters of the pepper plant

**Fig. 8:**
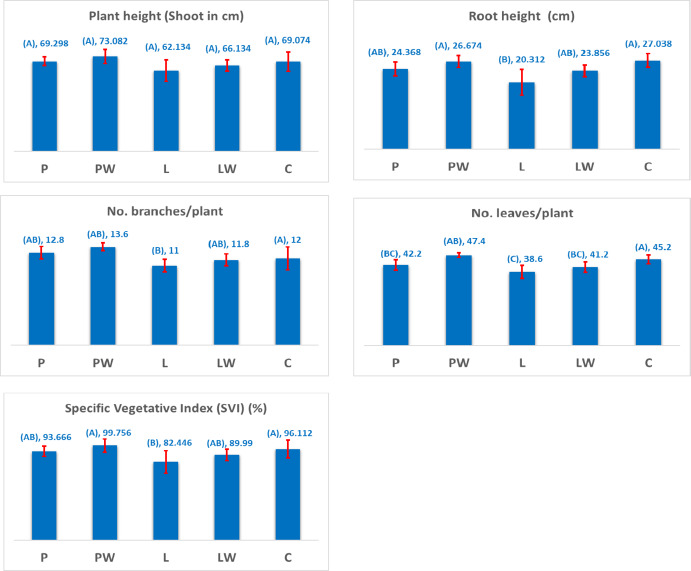
Variations in vegetative growth parameters of pepper plants across different types of biodegradable cultivating pots.

Vegetative growth parameters, including shoot height, root length, number of branches, leaves per plant, and Specific Vegetative Index (SVI), are depicted in Fig. 8. The most favorable characteristics were observed in plants cultivated in PW-pots. All properties, except plant height (shoot), exhibit significant variations based on pot types, as determined by one-way ANOVA. Pairwise comparisons using the Tukey method indicate that means not sharing a letter are significantly different at *P* < 0.05.

The mean height of pepper plants cultivated in different pot types was 69.29 cm for P-pots, 73.08 cm for PW-pots, 62.13 cm for L-pots, 66.13 cm for LW-pots, and 69.07 cm for commercial control (C-pot). Root height measurements were 24.36 cm, 26.67 cm, 20.31 cm, 23.85 cm, and 27.03 cm for P, PW, L, LW, and C-pots, respectively.

The number of branches per plant was recorded as 12.8, 13.6, 11.0, 11.8, and 12.0 for P, PW, L, LW, and C-pots, respectively. Similarly, the number of leaves per plant was measured at 42.2, 47.4, 38.6, 41.2, and 45.2 leaves per plant.

The Specific Vegetative Index (SVI) was 93.66 for P-pots, 99.75 for PW-pots, 82.44% for L-pots, 89.99% for LW-pots, and 96.11% for C-pots.

### Yield parameters of the pepper plant

**Fig. 9:**
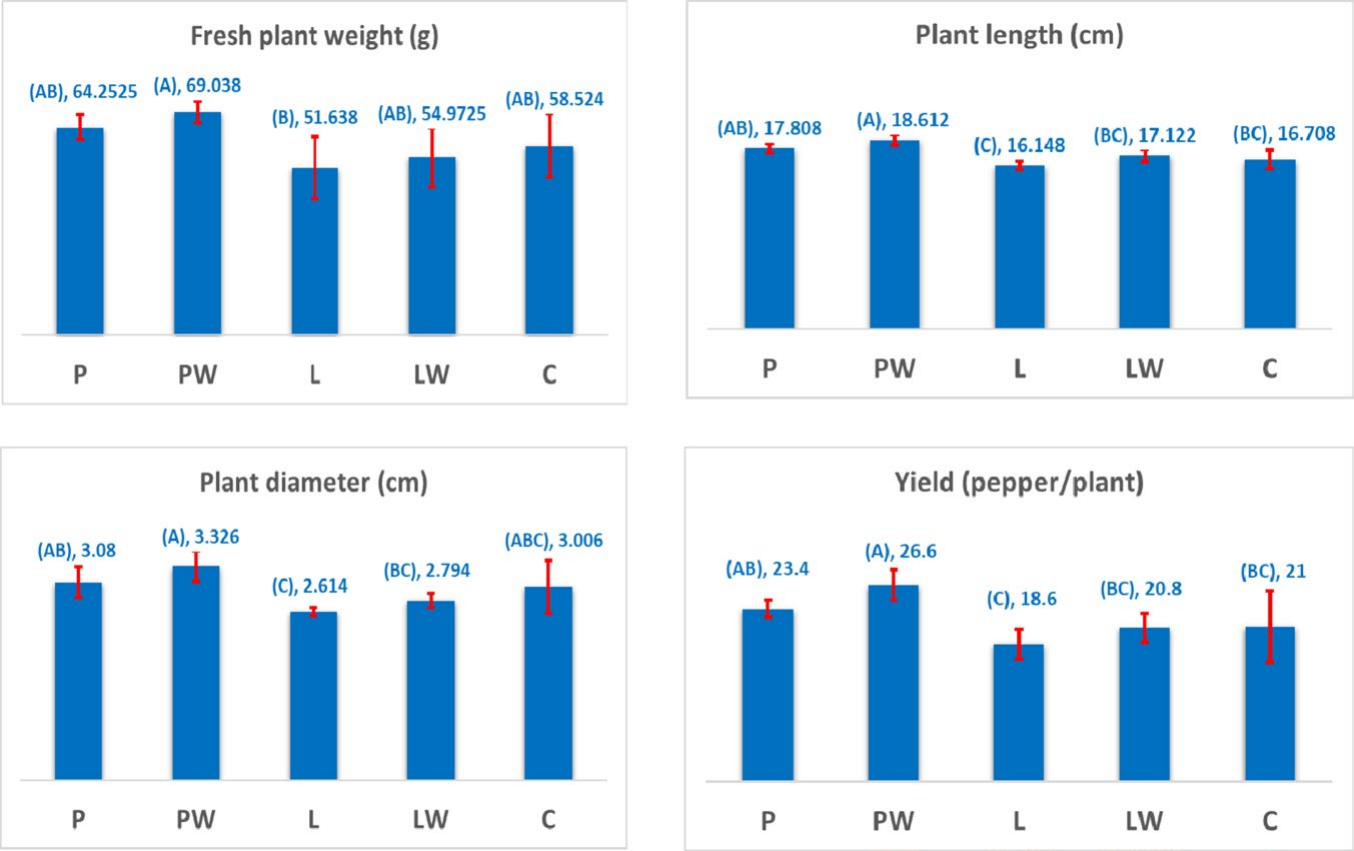
Variations in yield parameters of pepper plants across different types of biodegradable cultivating pots.

Yield parameters, including fresh plant weight, plant length, plant diameter, and the number of peppers per plant, are presented in Fig. 9. The highest yield performance was observed in plants cultivated in PW-pots. Statistical analysis using one-way ANOVA confirmed significant differences among pot types, with pairwise comparisons conducted via the Tukey method indicating that means not sharing a letter differ significantly.

The mean fresh weight of peppers was 64.25 g for P-pots, 69.03 g for PW-pots, 51.63 g for L-pots, 54.97 g for LW-pots, and 58.52 g for C-pots. Plant length measurements were recorded as 17.80 cm, 18.61 cm, 16.14 cm, 17.12 cm, and 16.70 cm for P, PW, L, LW, and C-pots, respectively.

The mean plant diameter was 3.08 cm for P-pots, 3.32 cm for PW-pots, 2.61 cm for L-pots, 2.79 cm for LW-pots, and 3.00 cm for C-pots. The number of peppers per plant was 23.4 for P-pots, 26.6 for PW-pots, 18.6 for L-pots, 20.8 for LW-pots, and 21.0 for C-pots.

Pepper yield per plant was highest in PW-pots, reaching 26.6 peppers, 13% higher than that observed in P-pots. This increase can be attributed to the reduced void content and improved nutrient availability, facilitated by the enhanced properties of the filler material.

These results are consistent with the findings of^19^, where pepper seedlings transplanted with biodegradable pots exhibited reduced transplant shock. In contrast, the control plants grown in polystyrene containers underwent conventional transplantation, requiring removal from the container before planting. The mean plant height, root development, and pepper yield per plant were higher in biodegradable pots compared to the control. This improvement can be attributed to the gradual release of nitrogen from the pot material during its degradation, enriching the growing medium and enhancing plant growth parameters.

### SWOT analysis of the biodegradable pots

SWOT analysis is a widely accepted tool in materials science for assessing the feasibility, strengths, and limitations of new material applications. While detailed cost analysis is typically conducted at the commercialization stage, SWOT provides an essential general strategic assessment, guiding further research and industrial adoption. Several scientific studies have employed SWOT analyses to evaluate the scalability, economic potential, and technological feasibility of biodegradable materials.

SWOT analysis was conducted for the biodegradable pot made from the palm wax as a favorable property from a plant growth perspective, sugarcane bagasse, compost, vermiculite, peat moss, and activated carbon:

### Strength

1. Environmental Friendliness: Incorporating biodegradable materials, including palm wax, sugarcane bagasse, compost, vermiculite, peat moss, and activated carbon, enhances the pot’s environmental profile, diminishing its overall environmental impact.
2. Biodegradability: engineered for natural decomposition, the pot contributes to soil enrichment and diminishes waste accumulation in landfills.
3. Versatility: balancing durability for pot cultivation with biodegradability renders it adaptable for various plants.
4. Nutrient-rich compost: Integration of compost, vermiculite, peat moss, and activated carbon potentially creates a nutrient-rich environment, fostering plant growth.
5. Appealing to eco-conscious consumers: Aligning with the growing preference for sustainable products, these biodegradable pots may appeal to environmentally conscious consumers and can be commercialized with further optimization.

### Weaknesses

1. Cost: Although detailed cost analysis is not the focus of this study, initial costs may be higher than traditional plastic pots. The pots aligned with the growing preference for sustainable products; these biodegradable pots may appeal to environmentally conscious consumers.
2. Durability: Compared to traditional plastics, biodegradable materials may lack durability, impacting pot longevity and suitability for specific plants or conditions.
3. Mass production challenges: Scaling up production may encounter hurdles in sourcing consistent and cost-effective raw materials, along with maintaining product quality.
4. Density and weight: reinforcement of fiber increases density and weight compared to plastic pots, necessitating a lighter alternative.
5. Shape: The incorporation of fibers results in a rough surface, which must be smoother to compete with the plastic’s surface.
6. Biodegradability compliance: Compliance with legislative standards for biodegradability might be insufficient.

### Opportunities

1. Growing green market: the rising demand for sustainable and eco-friendly products creates an opportunity for biodegradable pots in the market.
2. Transition to green ecological paradigm: the pot, enriched with various nutrients, reduces the need for fertilization and minimizes losses from runoff, contributing to cost-effective and environmentally conscious production.
3. Innovation Potential: Integration with smart agriculture (e.g., embedded nutrients or slow-release fertilizers).

### Threats

1. Market Competition: the deeply entrenched presence of traditional plastic pots poses a challenge in terms of cost, performance, and availability for biodegradable alternatives.
2. Regulatory Changes: evolving environmental regulations could impact the production and utilization of certain materials, affecting the feasibility of biodegradable pots.
3. Perceived Performance: consumer acceptance may hinge on the perceived effectiveness of biodegradable pots compared to traditional ones. Meeting performance expectations is crucial.
4. Supply Chain Disruptions: dependency on specific raw materials may expose the production process to supply chain vulnerabilities, influencing product availability and cost.

Briefly, despite offering environmental advantages, biodegradable pots face challenges such as cost, durability, and market competition in the horticultural industry. Strategic marketing, education, and partnerships are crucial for enhancing acceptance and adoption of these eco-friendly alternatives.

Future work should explore other fillers, binding agents, slow-release fertilizers and biodegradable enhancers to further optimize pot functionality and commercial viability.

## Conclusion

The study demonstrates the efficacy of mercerized sugarcane bagasse and other natural fillers in enhancing physical and mechanical properties of biodegradable cultivating pots. The incorporation of pretreatment techniques reduced water absorption by 35% and improved tensile strength by 4%, ensuring better structural integrity. Greenhouse trials confirmed the superior performance of treated pots, as evidenced by a 26% increase in the number of peppers per plant and an enhanced seedling vigor index of 99.75%.

A comparative analysis with commercial plastic pots revealed no significant differences in seed germination, as all pots exhibited a 100% germination rate. However, plant growth parameters, including plant height, root development, and yield, were notably improved in biocomposite pots. Specifically, plants cultivated in the optimized biocomposite pots exhibited greater biomass accumulation, increased branch and leaf counts, and a higher pepper yield per plant compared to those grown in commercial plastic pots. These results suggest that the gradual degradation of biocomposite pots enhances soil nutrient availability, mitigating transplant shock and fostering more robust plant development.

These findings emphasize the feasibility of using eco-friendly biocomposites in agriculture, offering an environmentally sustainable alternative to traditional plastic pots. Future research should explore the integration of slow-release fertilizers and advanced filler combinations to further enhance the functionality and marketability of biodegradable pots.

## Data Availability

The datasets used or analyzed during the current study are available from the corresponding author upon reasonable request.
